# The association of the platelet/high-density lipoprotein cholesterol ratio with self-reported stroke and cardiovascular mortality: a population-based observational study

**DOI:** 10.1186/s12944-024-02115-y

**Published:** 2024-04-24

**Authors:** Huifeng Zhang, Ying Xu, Yaying Xu

**Affiliations:** 1 Department of Cardiovascular, The First Affiliated Hospital, and College of Clinical Medicine of Henan University of Science and Technology, No. 24, Jinghua Road, Jianxi District, Luoyang City, Henan Province China; 2 Department of Hematology, The First Affiliated Hospital, and College of Clinical Medicine of Henan University of Science and Technology, Luoyang, China; 3 Department of Endocrinology, The First Affiliated Hospital, and College of Clinical Medicine of Henan University of Science and Technology, Luoyang, China

**Keywords:** High-density lipoprotein cholesterol, Platelet/high-density lipoprotein cholesterol ratio, Stroke, NHANES, Observational study

## Abstract

**Background:**

Previous studies have shown that the relationship between high-density lipoprotein cholesterol (HDL-C) and stroke is controversial, and the association between the platelet/high-density lipoprotein cholesterol ratio (PHR), a novel marker for inflammation and hypercoagulability states, and stroke has not been established.

**Methods:**

This study presents an analysis of cross-sectional data from the 2005–2018 National Health and Nutrition Examination Survey (NHANES). Stroke history, HDL-C levels, and platelet counts were obtained during cross-sectional surveys. The PHR was calculated as the ratio of the number of platelets to HDL-C concentration. Weighted logistic regression was used to assess the associations of HDL-C and the PHR with stroke. Nonlinearity of this relationship was determined through restricted cubic splines (RCSs) and two-piecewise linear regression for identifying inflection points. Furthermore, Cox regression was utilized to prospectively analyze the associations of the PHR and HDL-C concentration with cardiovascular disease (CVD) mortality in stroke survivors.

**Results:**

A total of 27,301 eligible participants were included in the study; mean age, 47.28 years and 50.57% were female, among whom 1,040 had a history of stroke. After full adjustment, the odds ratio (OR) of stroke associated with a per standard deviation (SD) increase in the PHR was estimated at 1.13 (95% confidence interval (CI): 1.03 − 1.24, *P* = 0.01), and the OR of stroke associated with a per SD increase in HDL-C was 0.95 (95% CI: 0.86–1.05, *P* = 0.30). The RCS indicated a nonlinear relationship for both variables (*P*_PHR_ = 0.018 and *P*_HDL-C_ = 0.003), and further piecewise linear regression identified inflection points at PHR = 223.684 and HDL-C = 1.4 mmol/L. Segmental regression indicated that in the PHR ≥ 223.684 segment, the estimated OR of stroke associated with a per-SD increase in the PHR was 1.20 (95% CI: 1.09 − 1.31, *P* < 0.001), while the association of stroke with HDL-C was not significant before or after the inflection point (*P* > 0.05). Furthermore, Cox regression and RCS showed that a per-SD increase in the PHR was linearly associated with a greater risk of CVD mortality among stroke survivors (HR: 1.14, 95% CI: 1.06 − 1.22, *P* < 0.001; nonlinear, *P* = 0.956), while HDL-C was not significantly associated with CVD mortality.

**Conclusion:**

The association between the PHR and stroke incidence exhibited a significant threshold effect, with an inflection point at 223.684. A PHR exceeding 223.684 was positively associated with stroke, while the association between HDL-C and stroke was not significant. Additionally, the PHR was positively and linearly associated with CVD mortality among stroke survivors.

**Supplementary Information:**

The online version contains supplementary material available at 10.1186/s12944-024-02115-y.

## Introduction

Stroke is the second leading cause of death globally, accounting for approximately 9% of annual deaths and imposing a significant burden on individual health, families, and society as a whole [1]. Factors such as blood pressure, blood glucose levels, physical activity, obesity, and diet are closely associated with the occurrence of stroke [2, 3]. Studies indicate that stroke is largely preventable, and early identification and management of modifiable risk factors are crucial for stroke prevention [4, 5].

High-density lipoprotein cholesterol (HDL-C) promotes the efflux of dietary cholesterol through the reverse cholesterol transport pathway and exhibits anti-inflammatory and antioxidant effects [6]. However, based on current epidemiological evidence, the relationship between HDL-C and stroke incidence appears to be uncertain, with reports of no significant association [7], an inverse linear relationship [8–10], a U-shaped association [11], and increased risks of cardiovascular and cerebrovascular mortality associated with very high levels of HDL-C (≥ 3.0 mmol/L) [12–15]. Importantly, this relationship remains inconclusive in the general population of the United States. Platelets are small blood cell fragments known for their ability to form clots to prevent bleeding; however, these clots can also lead to heart attacks and strokes [16]. HDL-C possesses antiplatelet, antithrombotic, and anti-inflammatory properties [17–19]. The platelet/high-density lipoprotein cholesterol ratio (PHR) was first proposed by Jialal et al. and is considered an effective biomarker for predicting metabolic syndrome (MetS) [20]. Since the introduction of the PHR, studies by Ni et al. have shown a positive nonlinear correlation between the PHR and the risk of kidney stones [21]. Research by Lu et al. [22] supports the use of the PHR as an effective marker for nonalcoholic fatty liver disease and liver fibrosis. A large meta-analysis in the past indicated that MetS is associated with a twofold increase in cardiovascular outcomes and a 1.5-fold increase in all-cause mortality [23]. Platelets and HDL-C are both sensitive indicators of stroke, and the direct cause of elevated platelet levels is the formation of blood clots, while HDL-C has antiplatelet and other antithrombotic effects [20, 21]. A study by Jialal et al. [20] also revealed a significant correlation between the PHR and high triglyceride and coagulation factor levels, indicating that the PHR may also reflect a state of disrupted coagulation balance in the body, which could serve as a theoretical basis for its association with hemorrhagic stroke.

In this context, the ratio of platelets to HDL-C may be a valuable diagnostic indicator for stroke. In this study, cross-sectional data from the National Health and Nutrition Examination Survey (NHANES) and death certificate data linked to the National Death Index (NDI) were analyzed to investigate the associations of the PHR and HDL-C with the odds of stroke and cardiovascular disease (CVD) mortality among stroke survivors.

## Methods

### Data source

**Fig. 1:**
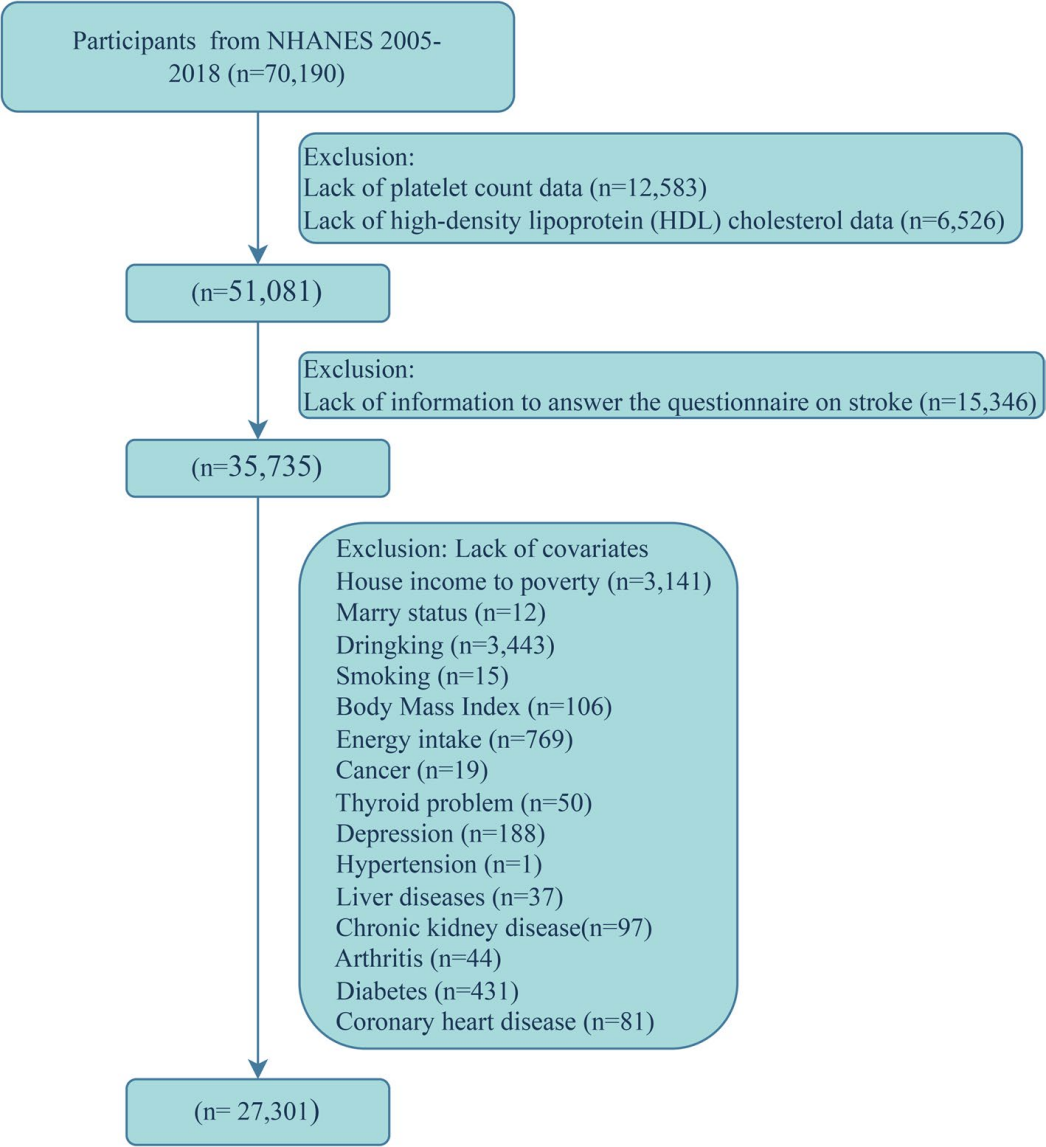
Flow chart for inclusion of participants

This cross-sectional study investigated participants from NHANES surveys conducted between 2005 and 2018, spanning a total of seven cycles. The NHANES utilizes a complex multistage probability sampling design in which data is collected through face-to-face interviews, physical examinations, and laboratory tests. The survey obtained ethical approval from the Institutional Review Board of the National Center for Health Statistics, and all participants provided written informed consent. The details are available at https://www.cdc.gov/nchs/nhanes/index.htm. Among the 70,190 participants across the seven cycles, those with the following missing data were sequentially excluded: platelet count (PC) in 12,583 individuals, HDL-C levels in 6,526 individuals, diagnosis data in 15,346 individuals, and covariate data in 8,434 participants. As a result, a total of 27,301 participants qualified for inclusion in the study (Fig. 1).

### PHR

During the cross-sectional survey, PC (1000 cells/μL) and HDL-C (mmol/L) were biochemical parameters obtained from blood samples collected from participants at the Mobile Examination Center. The PHR was calculated as the ratio of PC to HDL-C [20]. Due to the nonnormal distribution of PHR values, a standardized transformation (z-score) was performed.

### Stroke

Data on stroke history were acquired from the NHANES questionnaire [24, 25]. The information "Has a doctor or other health professional ever told you that you had a stroke?" from the Medical Conditions Questionnaire was utilized to ascertain the history of stroke, which included both hemorrhagic and ischemic strokes. Of particular note, patients may have had either clinical or imaging-based stroke, and the survey response was not specific to clinical stroke.

### Confirming the death status

The NDI provides death certificate data. The NHANES survey data were linked to the NDI data using probabilistic matching to ascertain participants' vital status. Participants for whom NDI matching was unsuccessful were presumed to be alive [26, 27]. The follow-up duration was calculated in years based on the time interval between the date of death or December 31, 2019 (whichever came first) and the recruitment date. Mortality related to CVD was determined based on the guidelines of the Tenth Revision of the International Statistical Classification of Diseases and Related Health Problems and included diseases of the heart (I00-I09, I11, I13, I20-I51) and cerebrovascular diseases (I60-I69).

### Covariates

In this study, confounding factors were determined based on previous literature and peer feedback [11]. Demographic characteristics of age (continuous), sex (male/female), race/ethnicity (Mexican–American, non-Hispanic black, non-Hispanic white, other Hispanic, other race—including multiracial), educational level (less than college, college, and above), and marital status (divorced/separated/widowed, married/cohabitating, never married) were self-reported at the time of the questionnaire. Additionally, the ratio of household income to poverty level (< 1.3, 1.3 − 3.5, > 3.5) was considered. Lifestyle and physical measures included alcohol consumption (never, former, current), smoking status (never, former, current), total dietary energy intake (categorized into high and low groups based on the median value), and body mass index (BMI), which was defined as weight in kilograms divided by the square of height in meters (kg/m^2^). Comorbidities included coronary heart disease (CHD), cancer, liver disease, chronic kidney disease (CKD) (diagnosed as A2, G3a, and above) [28], hypertension, diabetes mellitus (DM) (yes, no, impaired fasting glucose, impaired glucose tolerance), arthritis, thyroid disorders, and depression (determined by Patient Health Questionnaire-9, cutoff = 10) [29].

### Statistical analysis

The official documentation of the NHANES recommends that data analysis should be weighted (NHANES Tutorials—Weighting Module (cdc.gov)). In this study, the selected weight was the laboratory examination weight (1/7 * WTMEC2YR), as the important variables in this study were the PC and HDL-C concentration obtained from laboratory tests. To account for the complex sampling method involved in the NHANES database, continuous variables were compared using the Student's t test or Wilcoxon rank-sum test, and the results are presented as the means (standard error) or medians (interquartile range). Categorical variables were analyzed using the chi-square test, and the results are presented as counts (n) and percentages (%). To meet statistical requirements and facilitate interpretation, PHR or HDL-C were included in weighted multivariable logistic regression and Cox proportional hazards models as standardized z scores or divided by 100, estimating the associations between exposures and outcome risks as odds ratios (ORs), hazard ratios (HRs), and 95% confidence intervals (CIs). Multiple models were constructed as follows: Model 0, unadjusted; Model 1, adjusted for sex, age, race, and education; Model 2, further adjusted for marital status, household income-to-poverty ratio, alcohol consumption, smoking status, and BMI; Model 3, further adjusted for comorbidities, including coronary heart disease, hypertension, diabetes, depression, thyroid disorders, liver disease, arthritis, tumors, and CKD. In the survival analysis models, due to the small number of CVD-specific deaths among stroke survivors, least absolute shrinkage and selection operator (LASSO) regression was employed to select important covariates and simplify the model. Collinearity was assessed using the variance inflation factor (VIF); all the variance inflation factors (VIFs) were less than 10 in this study. The proportional hazards assumption was tested using Schoenfeld residuals. Subgroup analyses were conducted, and interactions between covariates and PHR were explored using likelihood ratio tests. Restricted cubic splines (RCSs) with three knots (10th, 50th, 90th percentiles) were used to assess the exposure-dose relationship between continuous PHR or HDL-C and stroke or stroke survivor CVD mortality risk. Additionally, two sensitivity analyses were performed: 1) additionally adjusted for antiplatelet medications (including aspirin, dipyridamole, cilostazol, clopidogrel, ticlopidine) and statins (atorvastatin, simvastatin, fluvastatin, lovastatin, nystatin, pravastatin, rosuvastatin, simvastatin) [30] and 2) without regard to sampling weights.

All the statistical analyses were conducted using R software version 4.3.1 (R Foundation for Statistical Computing). The "survey" package was utilized for weighted regression analysis; the "rms" package was employed to construct the RCS regression; the "segmented" package was used to fit a two-piecewise linear regression to determine the inflection point; the "glmnet" package was utilized for implementing LASSO regression for feature selection; and the "car" package was used to calculate variance inflation factors. A two-tailed *P* value < 0.05 was considered statistically significant.

## Results

### Population characteristics

After applying strict exclusion criteria, a total of 27,301 participants were enrolled in the study (Fig. 1), among whom 1,040 had a history of stroke, with a weighted prevalence of 2.83%; the average age was 47.28 years and 50.57% were female (Table 1). Compared to nonstroke participants, the following were characteristic of those with stroke: older age, a greater proportion of females, significant differences in racial composition, lower educational levels, more likely to be divorced/separated/widowed, poorer economic conditions, higher rates of past smoking and alcohol consumption, higher energy intake, higher BMI, and a greater incidence of chronic diseases and depression (Table 1). On the other hand, during a mean follow-up period of 6.58 years, 367 deaths were identified among the 1,040 stroke survivors, with 139 deaths attributed to CVD; the final survival analysis model included 812 individuals. The following were characteristic of the individuals who died from CVD-specific causes: older age; more likely to be male; lower economic status; higher rates of past alcohol consumption, lower BMI, and lower energy intake; and significantly greater rates of cancer, CKD, hypertension, and CHD (Supplementary Table 1).

**Table 1:** Weighted characteristics of the eligible 27,301 participants in the analysis of PHR and HDL-C with the odds of stroke

Characteristics	Total (*N* = 27,301)	Healthy (*N* = 26,261)	Stroke (*N* = 1,040)	*P* value
PHR, median(Q1,Q3)	**183.19(141.38,236.73)**	**183.33(141.52,236.73)**	**178.83(136.65,238.55)**	**0.35**
Platelet (1000 cells/μL), median(Q1,Q3)	**241.00(206.00,285.00)**	**242.00(206.00,285.00)**	**233.00(192.00,278.00)**	**0.002**
HDL-C (mmol/L), median(Q1,Q3)	**1.32(1.09,1.60)**	**1.32(1.09,1.60)**	**1.29(1.03,1.60)**	**0.02**
Age (year), Mean (S.E)	**47.28(0.25)**	**46.79(0.25)**	**64.13(0.59)**	** < 0.0001**
Sex, n (%)				**0.02**
Female	**13,619(50.57)**	**13,094(50.42)**	**525(55.52)**	
Male	**13,682(49.43)**	**13,167(49.58)**	**515(44.48)**	
Race, n (%)				** < 0.0001**
Mexican American	**4160(7.94)**	**4066(8.04)**	**94(4.35)**	
Non-Hispanic Black	**5585(10.15)**	**5318(10.06)**	**267(13.44)**	
Non-Hispanic White	**12,415(70.22)**	**11,858(70.15)**	**557(72.86)**	
Other Hispanic	**2467(5.00)**	**2402(5.06)**	**65(2.95)**	
Other Race—Including Multi-Racial	**2674(6.68)**	**2617(6.69)**	**57(6.40)**	
Education attainment, n (%)				** < 0.0001**
Less than college	**12,508(37.69)**	**11,893(37.22)**	**615(53.84)**	
College or higher	**14,793(62.31)**	**14,368(62.78)**	**425(46.16)**	
Marital status, n (%)				** < 0.0001**
Never married	**4861(17.54)**	**4780(17.86)**	**81(6.55)**	
Divorced/separated/widowed	**6015(18.21)**	**5615(17.78)**	**400(32.79)**	
Married/living with a partner	**16,425(64.25)**	**15,866(64.36)**	**559(60.67)**	
Poverty, n (%)				** < 0.0001**
< 1.3	**8256(20.06)**	**7845(19.74)**	**411(31.02)**	
1.3–3.5	**10,339(35.54)**	**9893(35.26)**	**446(45.14)**	
> 3.5	**8706(44.40)**	**8523(45.00)**	**183(23.84)**	
Alcohol status, n (%)				** < 0.0001**
Never	**3647(10.28)**	**3483(10.16)**	**164(14.25)**	
Former	**4455(13.23)**	**4101(12.72)**	**354(30.66)**	
Now	**19,199(76.49)**	**18,677(77.12)**	**522(55.09)**	
Smoke, n (%)				** < 0.0001**
Never	**14,872(54.90)**	**14,463(55.28)**	**409(41.54)**	
Former	**6747(25.03)**	**6355(24.72)**	**392(35.88)**	
Now	**5682(20.07)**	**5443(20.00)**	**239(22.58)**	
Body Mass Index (BMI), Mean (S.E)	**29.08(0.08)**	**29.04(0.08)**	**30.37(0.29)**	** < 0.0001**
Total energy intake (Kcal) Mean (S.E)	**2195.67(8.93)**	**2205.53(8.68)**	**1856.94(41.05)**	** < 0.0001**
Cancer, n (%)	**2581(9.92)**	**2355(9.59)**	**226(21.29)**	** < 0.0001**
Thyroid problem, n (%)	**2811(10.93)**	**2602(10.58)**	**209(22.70)**	** < 0.0001**
Liver diseases, n (%)	**1057(3.47)**	**982(3.37)**	**75(6.90)**	** < 0.0001**
Arthritis, n (%)	**7472(25.76)**	**6877(24.87)**	**595(56.24)**	** < 0.0001**
Diabetes, n (%)				** < 0.0001**
DM	**5051(13.83)**	**4623(13.15)**	**428(36.95)**	
IFG	**1278(4.82)**	**1230(4.79)**	**48(5.86)**	
IGT	**1099(3.62)**	**1059(3.63)**	**40(3.12)**	
No	**19,873(77.74)**	**19,349(78.43)**	**524(54.06)**	
Depression, n (%)	**2336(7.47)**	**2151(7.18)**	**185(17.18)**	** < 0.0001**
Coronary heart disease, n (%)	**1099(3.36)**	**922(2.94)**	**177(18.05)**	** < 0.0001**
CKD, n (%)	**4788(13.90)**	**4315(13.11)**	**473(41.01)**	** < 0.0001**
Hypertension, n (%)	**11,593(37.60)**	**10,747(36.42)**	**846(78.39)**	** < 0.0001**
Antiplatelets, n (%)	**948(2.46)**	**693(1.87)**	**255(22.72)**	** < 0.0001**
Statin drugs, n (%)	**5147(16.75)**	**4618(15.74)**	**529(51.37)**	** < 0.0001**

*Abbreviation*: *HDL-C* High-density lipoprotein cholesterol, *PHR* Platelet-to-high-density lipoprotein cholesterol ratio, *CKD* Chronic kidney disease, *IFG* Impaired fasting glycaemia, *IGT* Impaired glucose tolerance, *DM* Diabetes, *SE* Standard error

### Estimation of the associations of the PHR and HDL-C concentration with stroke incidence

The results of weighted multivariable logistic regression are shown in Table 2. According to the crude model (Model 0), a per-SD increase in the PHR [OR (95% CI): 1.03 (0.93, 1.15), *P* = 0.055] and HDL-C [OR (95% CI): 0.92 (0.83, 1.01), *P* = 0.08] was not associated with stroke. After fully adjusting for stroke risk factors (Model 3), the OR of stroke associated with a per-SD increase in the PHR was 1.13 (95% CI: 1.03 − 1.24, *P* = 0.01) and that associated with a per-SD increase in HDL-C was 0.95 (95% CI: 0.86 − 1.05, *P* = 0.30).

**Table 2:** Weighted multivariate Logistic regression analysis for the association of stroke with PHR and HDL-C

PHR		Model 0		Model 1		Model 2		Model 3	
Overall		OR (95% CI)	*P*	OR (95% CI)	*P*	OR (95% CI)	*P*	OR (95% CI)	*P*
	Per SD increase	1.03(0.93,1.15)	0.55	1.23(1.13,1.33)	< 0.0001	1.17(1.07,1.27)	< 0.001	1.13(1.03,1.24)	0.01
	Per 100 increase	1.04(0.91,1.19)	0.55	1.29(1.16,1.42)	< 0.0001	1.21(1.09,1.34)	< 0.001	1.16(1.04,1.30)	0.01
PHR < 223.684	Per SD increase	0.87(0.78,0.97)	0.01	1.02(0.92,1.14)	0.65	0.98(0.89,1.09)	0.77	0.97(0.87,1.08)	0.53
	Per 100 increase	0.70(0.54,0.91)	0.01	1.06(0.81,1.39)	0.65	0.96(0.74,1.26)	0.77	0.92(0.70,1.20)	0.53
PHR ≥ 223.684	Per SD increase	1.22(1.12,1.33)	< 0.0001	1.26(1.14,1.38)	< 0.0001	1.22(1.12,1.34)	< 0.0001	1.20(1.09,1.31)	< 0.001
	Per 100 increase	1.31(1.17,1.47)	< 0.0001	1.36(1.19,1.55)	< 0.0001	1.32(1.16,1.49)	< 0.0001	1.27(1.13,1.44)	< 0.001
HDL-C		Model 0		Model 1		Model 2		Model 3	
Overall		OR (95% CI)	*P*	OR (95% CI)	*P*	OR (95% CI)	*P*	OR (95% CI)	*P*
	Per SD increase	0.92(0.83,1.01)	0.08	0.79(0.71,0.88)	< 0.0001	0.90(0.81,1.01)	0.07	0.95(0.86,1.05)	0.30
	Per 1 increase	0.81(0.65,1.02)	0.08	0.56(0.44,0.73)	< 0.0001	0.78(0.59,1.02)	0.07	0.88(0.70,1.12)	0.30
HDL-C ≥ 1.4 mmol/L	Per SD increase	0.87(0.77,0.97)	0.01	0.79(0.70,0.90)	< 0.001	0.86(0.75,0.97)	0.02	0.91(0.80,1.03)	0.14
	Per 1 increase	0.46(0.25,0.83)	0.01	0.29(0.15,0.55)	< 0.001	0.43(0.22,0.87)	0.02	0.60(0.30,1.20)	0.14
HDL-C ≥ 1.4 mmol/L	Per SD increase	1.09(0.97,1.22)	0.14	1.00(0.88,1.14)	0.99	1.06(0.94,1.21)	0.35	1.04(0.92,1.19)	0.53
	Per 1 increase	1.28(0.92,1.79)	0.14	1.00(0.68,1.46)	0.99	1.20(0.82,1.74)	0.35	1.13(0.77,1.66)	0.53

Model 0: Not adjusted

Model 1: Adjusted for age, sex, education attainment, and ethnicity

Model 2: Further adjusted for marital status, poverty-income ratio, smoking, and drinking status, BMI, and total energy intake based on Model 1

Model 3: Further adjusted for arthritis, thyroid problems, cancer, diabetes, depression, hypertension, liver diseases, CHD, and CKD on Model 2

*Abbreviations*: *HDL-C* High-density lipoprotein cholesterol, *PHR* Platelet-to-high-density lipoprotein cholesterol ratio, *BMI* Body mass index, *CHD* Coronary heart disease, *CKD* Chronic kidney disease, *SD* Standard deviation, *OR* Odds ratio, *CI* Confidence interval

**Fig. 2:**
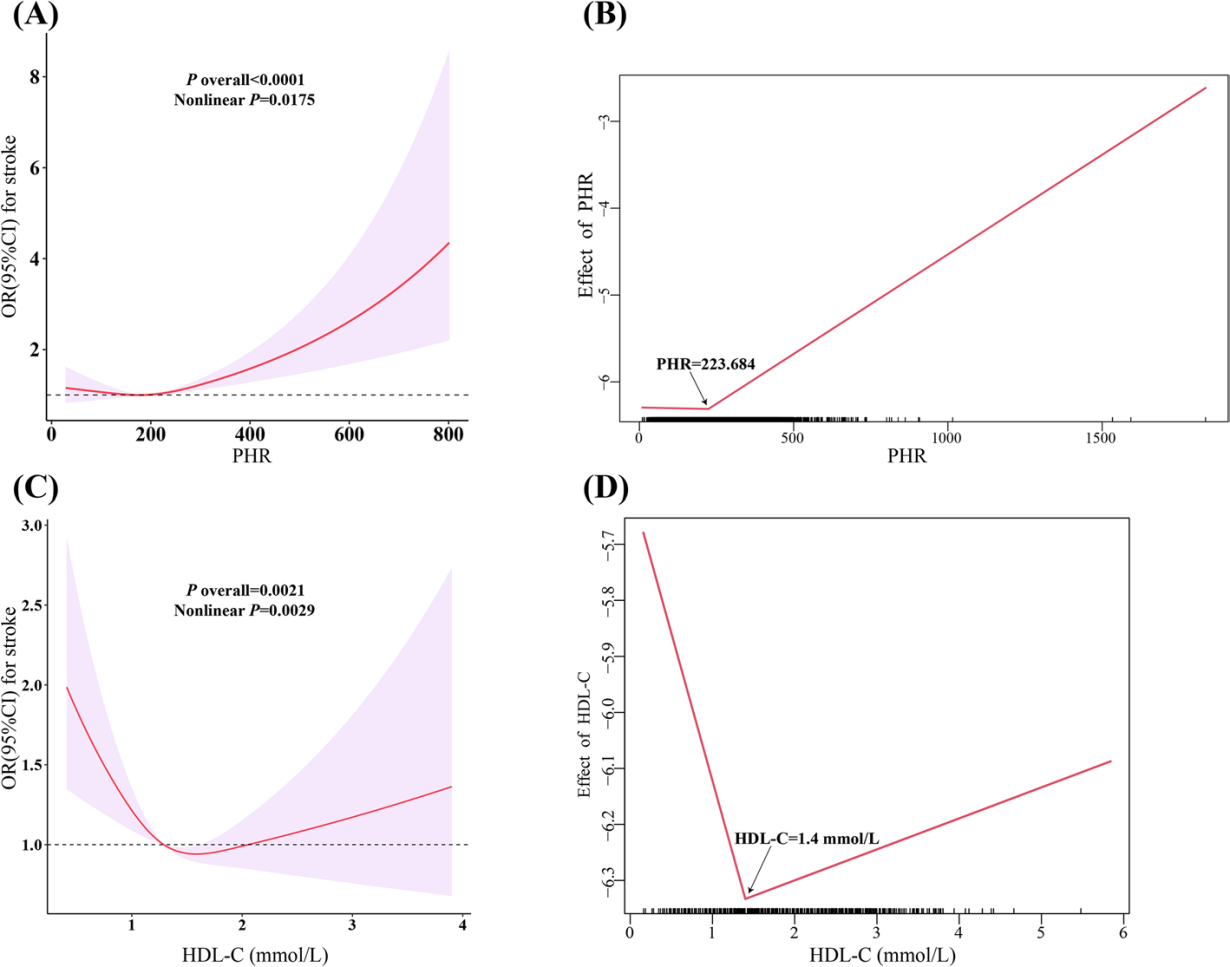
Dose–response relationship and the two-piecewise linear regression of PHR and HDL-C with stroke. Legend: Three knots (10th, 50th, 90th percentiles) were selected for fitting the restricted cubic spline model, and the median value of PHR (184.375) and HDL-C (1.29 mmol/L) served as the reference point. All models were adjusted for age, sex, ethnicity, education, marital status, poverty-income ratio, total energy intake, BMI, smoking, drinking, arthritis, thyroid problems, cancer, diabetes, depression, hypertension, liver diseases, CHD, and CKD. Abbreviation: HDL-C, high-density lipoprotein cholesterol; PHR, platelet-to-high-density lipoprotein cholesterol ratio; BMI, body mass index; CHD, coronary heart disease; CKD, chronic kidney disease; OR, odds ratio; CI, confidence interval

The RCS showed that both the PHR and HDL-C had a nonlinear relationship with stroke (*P* = 0.018 and 0.003, respectively) (Fig. 2A and C). Further piecewise linear regression revealed inflection points at PHR = 223.684 and HDL-C = 1.4 mmol/L (Fig. 2B and D). Table 2 presents the results of segmented regression before and after the inflection points. In the PHR < 223.684 segment, o significant association between the PHR and stroke was observed (OR: 0.97, 95% CI: 0.87 − 1.08, *P* = 0.53); in the PHR ≥ 223.684 segment, the OR of stroke associated with a per SD increase in the PHR was 1.20 (95% CI: 1.09 − 1.31, *P* < 0.001). However, the association between stroke and HDL-C was not significant in either segment before or after the inflection point (*P* > 0.05) (Table 2).

### Estimation of the associations of the PHR and HDL-C concentration with the risk of CVD mortality and stroke survival

**Fig. 3:**
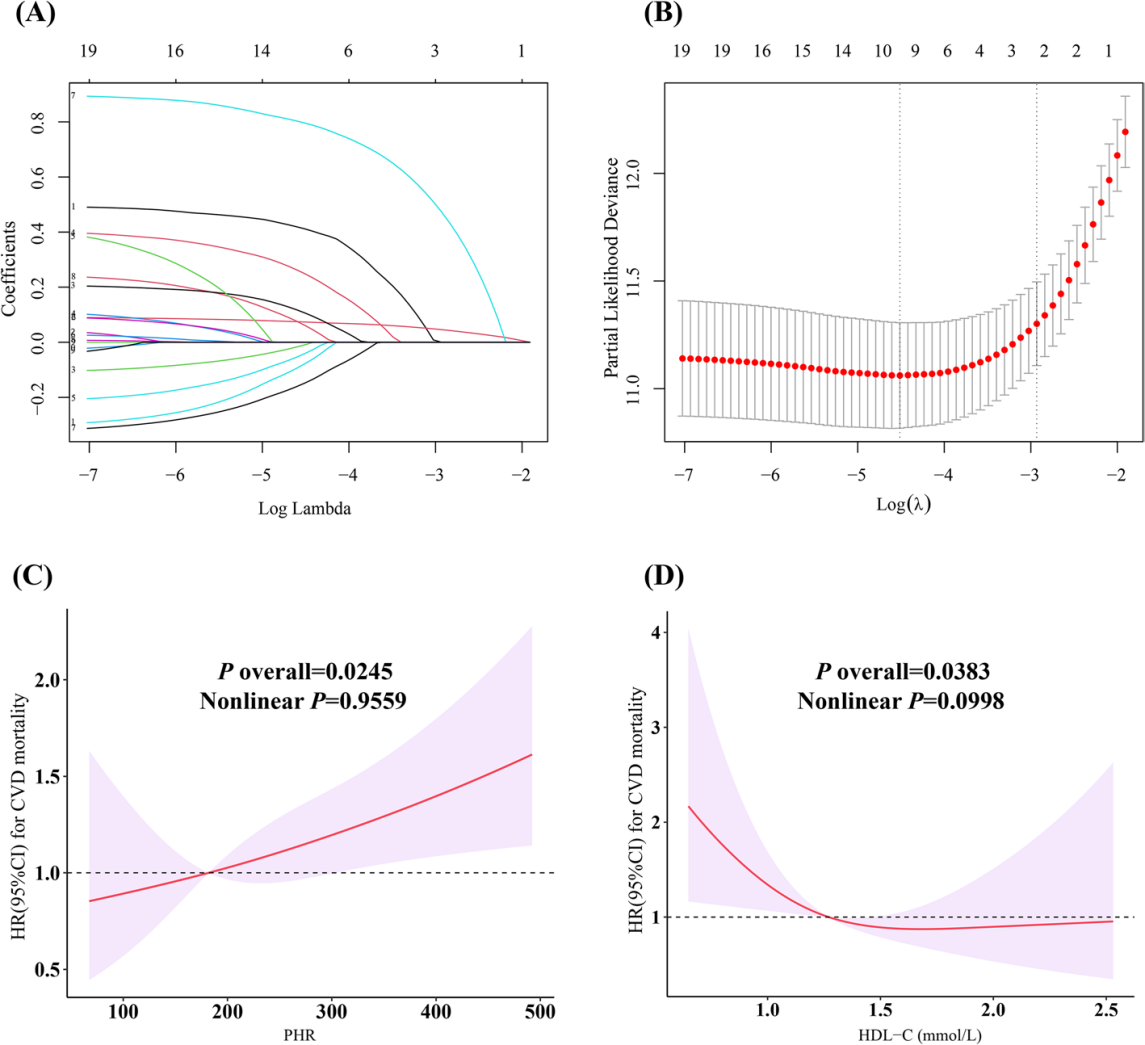
LASSO regression analysis of the 19 covariates associated with CVD mortality and Dose–response relationship between PHR and CVD mortality among stroke survivals. Legend: **A** The screening path corresponds to 19 covariates that contribute to CVD mortality among stroke survivors. **B** The association between the log-transformed λ and Partial Likelihood Deviance for CVD mortality among stroke survivors. The red dashed line and its error bars represent the average Partial Likelihood Deviance value and the corresponding 95% CI. The left black dashed line represents the optimal λ value obtained when calculating the minimum Partial Likelihood Deviance, while the right black dashed line represents the λ value of the simplest model obtained at one standard error from the minimum Partial Likelihood Deviance. **C** Dose–response relationship between PHR and CVD mortality among stroke survivals. **D** Dose–response relationship between HDL-C and CVD mortality among stroke survivals. Three knots (10th, 50th, 90th percentiles) were selected for fitting the restricted cubic spline model, and the median value of PHR (181.91) and HDL-C (1.27 mmol/L) served as the reference point, and models were adjusted for age, sex, marital status, smoking, drinking, thyroid problems, cancer, CHD and CKD. Abbreviation: HDL-C, high-density lipoprotein cholesterol; PHR, platelet-to-high-density lipoprotein cholesterol ratio; CVD, cardiovascular disease; CHD, coronary heart disease; CKD, chronic kidney disease; LASSO, least absolute shrinkage, and selection operator; HR, hazard ratio; CI, confidence interval

Considering that this study included only 139 cases of CVD-specific deaths, LASSO regression was used for variable selection and identified a set of important variables, including sex, age, marital status, alcohol consumption, smoking status, thyroid problems, and cancer, CHD, and CKD diagnoses, totaling 9 factors (Fig. 3A and B). A multivariate Cox regression model was constructed based on these variables. Schoenfeld residual tests indicated that all models met the proportional hazards assumption. As shown in Table 3, weighted Cox regression revealed that the PHR showed no significant association with CVD mortality in the unadjusted model (Model 0) (HR: 1.07, 95% CI: 0.93 − 1.23, *P* = 0.33); however, after stepwise adjustment for demographic characteristics, lifestyle, and comorbidities, the PHR significantly predicted the risk of CVD mortality. In the completely adjusted model (Model 3), a per SD increase in the PHR was associated with a greater risk of CVD mortality among stroke survivors (HR: 1.14, 95% CI: 1.06 − 1.22, *P* < 0.001), and the RCS fit a strong positive linear dose‒response relationship between the PHR and CVD mortality (*P* = 0.956) (Fig. 3C). Although the RCS regression showed a potential linear relationship of HDL-C to CVD mortality (*P* = 0.0998, nonlinear) (Fig. 3D), it was not associated with CVD mortality in any of the Cox regression models (all *P* > 0.10) (Table 3).

**Table 3:** Weighted multivariate Cox regression analysis for the association of CVD mortality with PHR and HDL-C

PHR	Model 0		Model 1		Model 2		Model 3	
	HR (95% CI)	*P*	HR (95% CI)	*P*	HR (95% CI)	*P*	HR (95% CI)	*P*
Per SD increase	1.07(0.93,1.23)	0.33	1.11(1.03,1.19)	0.01	1.10(1.02,1.18)	0.01	1.14(1.06,1.22)	< 0.001
Per 100 increase	1.07(0.94,1.21)	0.33	1.10(1.03,1.18)	0.01	1.10(1.02,1.17)	0.01	1.13(1.06,1.21)	< 0.001
HDL-C	Model 0		Model 1		Model 2		Model 3	
	HR (95% CI)	*P*	HR (95% CI)	*P*	HR (95% CI)	*P*	HR (95% CI)	*P*
Per SD increase	0.86(0.70,1.06)	0.17	0.82(0.65,1.04)	0.10	0.84(0.66,1.06)	0.14	0.83(0.66,1.05)	0.13
Per 1 increase	0.70(0.42,1.16)	0.17	0.63(0.36,1.10)	0.10	0.66(0.38,1.15)	0.14	0.65(0.37,1.13)	0.13

Model 0: Not adjusted

Model 1: Adjusted for age and sex

Model 2: Further adjusted for marital status, smoking, and drinking status based on Model 1

Model 3: Further adjusted for thyroid problems, cancer, CHD, and CKD based on Model 2

*Abbreviations*: *HDL-C* High-density lipoprotein cholesterol, *PHR* Platelet-to-high-density lipoprotein cholesterol ratio, *CVD* Cardiovascular disease, *CHD* Coronary heart disease, *CKD* Chronic kidney disease, *SD* Standard deviation, *HR* Hazard ratio, *CI* Confidence interval

### Subgroup analysis and sensitivity analysis

Subgroup analysis revealed that overall, the associations between PHR and stroke, as well as CVD mortality, were generally consistent across different segmentation intervals. When the PHR was > 223.684, a significantly stronger association between the PHR and stroke was observed in the noncancer population (*P* for interaction = 0.028) (Supplementary Figs. 1 and 2). In the sensitivity analysis, after additional adjustment for antiplatelets and statins or without weighting, the associations between the PHR and stroke, as well as CVD mortality among stroke survivors, remained robust (Supplementary Tables 2 and 3).

## Discussion

In this large-scale observational study, the results provided strong evidence supporting a significant association of the PHR with stroke, while HDL-C was not significantly associated with stroke. Additionally, a strong linear positive association between the PHR and CVD mortality was observed among stroke survivors; however, HDL-C was not associated with the risk of CVD mortality among stroke survivors. Furthermore, by combining RCS regression and two-piecewise linear regression, a PHR of 223.684 was determined to be the inflection point for the association between the PHR and the prevalence of stroke. Below this value, there was no significant association between stroke and increasing PHR; however, beyond this value, the odds of stroke sharply increased. In conclusion, the study findings suggest an association between the PHR and both the risk of stroke and CVD mortality among stroke survivors.

Although the mainstream view holds that HDL-C is beneficial for human health [31], studies have shown that active intervention therapy for HDL-C does not help reduce the risk of cardiovascular events [32–35]. In 2001, a study from the Atherosclerosis Risk in Communities cohort indicated that individuals in any of the other three quartiles of HDL-C did not show a significant change in overall risk of ischemic stroke compared to participants in the lowest quartile, even after stratification by sex [36]. Similarly, in 2007, Kurth et al. [37] conducted a cohort study involving over 28,000 women aged ≥ 45 years in the United States in which no significant association between HDL-C and ischemic stroke (HR: 0.78, 95% CI: 0.52–1.17) was observed after adjusting for other stroke risk factors. A prospective cohort study of Asian adults also revealed that HDL-C levels do not influence the risk of ischemic (HR: 0.90, 95% CI: 0.75 − 1.07) or hemorrhagic (HR: 0.89, 95% CI: 0.74 − 1.07) stroke [38]. In the present study, a similar trend was demonstrated, indicating no correlation between HDL-C and stroke according to both the overall and segmented regression analyses. On the other hand, a meta-analysis incorporating more than 10 randomized clinical trials indicated that drug interventions to increase HDL-C levels did not improve CVD mortality (OR: 0.99 [95% CI, 0.89 − 1.11]) or the odds of ischemic stroke (OR: 1.00 [95% CI, 0.78 − 1.30]) [35]. Furthermore, prospective evidence indicates that elevated HDL-C (≥ 3.0 mmol/L) may even increase the risk of all-cause mortality, cardiovascular mortality, and cerebrovascular mortality [12–15]. In this study, no significant association was found between HDL-C and CVD-specific mortality risk among stroke survivors.

Jialal et al. [20] suggested that the PHR could be a biomarker for the risk of atherosclerotic thrombosis; however, this finding needs further confirmation in additional studies. Our study contributes to the knowledge in this field. In the present study, when the PHR was > 223.684, the odds of stroke significantly increased, and a proportional dose‒response relationship between the PHR and CVD mortality risk was observed among stroke survivors. Mechanistically, the direct risk associated with an elevated PC is the formation of blood clots, while HDL-C has antiplatelet and other antithrombotic effects [20, 21]. Atherosclerosis is an important pathogenic mechanism for both ischemic and hemorrhagic stroke [39–41]. Reduced HDL-C levels impede effective cholesterol transport, leading to deposition within arterial walls and promoting the formation of atherosclerotic plaques [42] An elevated PC enhances blood coagulability, facilitating thrombus formation at sites of arterial injury and thereby contributing to the development of atherosclerotic lesions [43]. Research by Jialal et al. [20] demonstrated a significant correlation between the PHR and elevated triglycerides and coagulation factors, suggesting that an elevated PHR may reflect an imbalance in coagulation homeostasis. Ischemic stroke patients often exhibit coagulation abnormalities, with post-stroke complications frequently related to coagulation dysfunction [44]. In the current study, the PHR showed a linear positive correlation with cardiovascular-specific mortality among stroke survivors. On the other hand, small vessel disease is one of the mechanisms underlying ischemic stroke and is associated with elevated blood pressure [41], and hypertension also increases intravascular pressure, predisposing patients to vascular rupture and hemorrhagic stroke [45]. Several Mendelian randomization studies have established a positive causal relationship between an elevated PC and hypertension in European or Asian populations [46–48]. Platelets induce hypertension by increasing intracellular calcium concentrations in vascular smooth muscle cells, leading to vasoconstriction [49]; nitric oxide (NO) has hypotensive effects, but activated platelets generate reactive oxygen species (ROS), which inhibit NO activity [50–52]. Furthermore, despite recent studies revealing that HDL-C is not always a "good protein," the majority still support the notion that lower HDL-C predicts future hypertension [53–58]. As a ratio of PC to HDL-C, the PHR may mediate stroke occurrence through its effect on blood pressure. Additionally, systemic inflammation plays a critical role in stroke development [59]. Elevated levels of IL-1β, IL-6, and TNF-α are closely associated with stroke occurrence, while some lipid-lowering drugs, such as statins, have been shown to reduce stroke risk through their anti-inflammatory effects [60, 61]. Platelets serve as important markers for assessing systemic inflammation; conversely, HDL-C exhibits anti-inflammatory properties [17–19, 21]. Therefore, the association between the PHR and stroke may be interpreted from an inflammatory perspective. Furthermore, in the present study, interaction analysis showed that malignancy altered the association between the PHR and stroke incidence. This finding may be due to the fact that more than 30% of patients with solid malignant tumors have thrombocytosis, and pro-tumor growth cytokines within malignant tumor cells, such as IL-1, IL-3, and TNF-α, specifically stimulate platelet proliferation, resulting in an insignificant association between the PHR and stroke [62–64].

## Study strengths and limitations

To our knowledge, this is the first observational study to explore the relationship between the PHR and the prevalence of stroke among the adult population in the United States. This study comprehensively considered the risk factors for stroke, making the conclusions relatively reliable. Second, given that both HDL-C concentration and PC are routine indicators that are easy to obtain, they may have considerable potential in the diagnosis and prognosis assessment of stroke.

However, some limitations need to be considered. First, due to the cross-sectional design, causality in the relationship between the PHR, HDL-C concentration, and stroke cannot be inferred, necessitating further validation in more large-scale, prospective cohort studies. Additionally, due to sampling bias, the study results may not represent the general population. Second, the temporal relationship between the PHR and stroke risk is not known, and the history of stroke in this study was based on self-reports, which may not be accurate and cannot be generalized to the real-world incidence of clinical stroke. Third, HDL-C includes multiple HDL subgroups, each with different biological characteristics; however, in this study, data on the number and size of HDL subgroups or HDL particles were not obtained. Fourth, potential confounding factors were inevitably overlooked. Fifth, the types of stroke are indistinguishable in the NHANES; hence, this study represents the overall situation of stroke and cannot be generalized to specific subtypes. Sixth, stroke is related to platelet function. However, this study could not determine the functional parameters of platelets [65, 66]. Lastly, this study is based on a single measurement of HDL-C concentration and platelet count at the time of the cross-sectional survey, which may lead to potential bias.

## Conclusion

In conclusion, this study revealed an association between the PHR and stroke as well as between the PHR and the prognosis of stroke survivors. The PHR can serve as a potential biomarker for stroke, especially when the PHR is greater than 223.684. Additionally, the PHR has significant prognostic value for cardiovascular outcomes in stroke survivors.

### Supplementary Information

**Supplementary Material 1.**

## Data Availability

Data used for this study are available on the NHANES website: https://wwwn.cdc.gov/nchs/nhanes/.

## Abbreviations

BMI: Body mass index
CHD: Coronary heart disease
CI: Confidence interval
CKD: Chronic kidney disease
CVD: Cardiovascular disease
DM: Diabetes mellitus
HDL-C: High-density lipoprotein cholesterol
HR: Hazard ratios
IL-1: Interleukin-1
IL-1β: Interleukin-1 beta
IL-3: Interleukin-3
IL-6: Interleukin-6
LASSO: Least absolute shrinkage and selection operator
MetS: Metabolic syndrome
NDI: National death index
NHANES: National health and nutrition examination survey
NO: Nitric oxide
OR: Odds ratio
PC: Platelet count
PHQ-9: Patient health questionnaire-9
PHR: Platelet-to-high-density lipoprotein cholesterol ratio
RCS: Restricted cubic splines
ROS: Reactive oxygen species
SD: Standard deviation
TNF-α: Tumor necrosis factor-alpha
VIF: Variance inflation factor
