# Supplementary material for: The association of the platelet/high-density lipoprotein cholesterol ratio with self-reported stroke and cardiovascular mortality: a population-based observational study
Source: Lipids Health Dis. 2024 Apr 24;23:121. doi: 10.1186/s12944-024-02115-y (PMC11040779; doi:10.1186/s12944-024-02115-y)
Supplement: Supplementary file 1 — Supplementary Material 1. [file 12944_2024_2115_MOESM1_ESM.docx]

The association of the platelet/high-density lipoprotein cholesterol ratio with self-reported stroke and cardiovascular mortality: A population-based observational study

Huifeng Zhang^1†*^, Ying Xu^2†^, Yaying Xu^3†^

^†^: Equal contributions by the authors

^1^Department of Cardiovascular, The First Affiliated Hospital, and College of Clinical Medicine of Henan University of Science and Technology, Luoyang, China.

^2^Department of Hematology, The First Affiliated Hospital, and College of Clinical Medicine of Henan University of Science and Technology, Luoyang, China.

^3^Department of Endocrinology, The First Affiliated Hospital, and College of Clinical Medicine of Henan University of Science and Technology, Luoyang, China.

Address: No. 24, Jinghua Road, Jianxi District, Luoyang City, Henan Province, China

# Supplementary Figures

## Supplementary Figure 1: Forest plot of subgroup analysis and interaction tests for the association between PHR and the odds of stroke

**Figure legend:** All models adjusted for 18 risk factors other than stratification variables, and the significance of the interaction was determined by the likelihood ratio test.

**Abbreviation:** PHR, platelet-to-high-density lipoprotein cholesterol ratio; CKD, chronic kidney disease; IFG, impaired fasting glycaemia; IGT, impaired glucose tolerance; DM, diabetes; OR, odds ratio; CI, confidence interval; *P* int, *P* interaction.
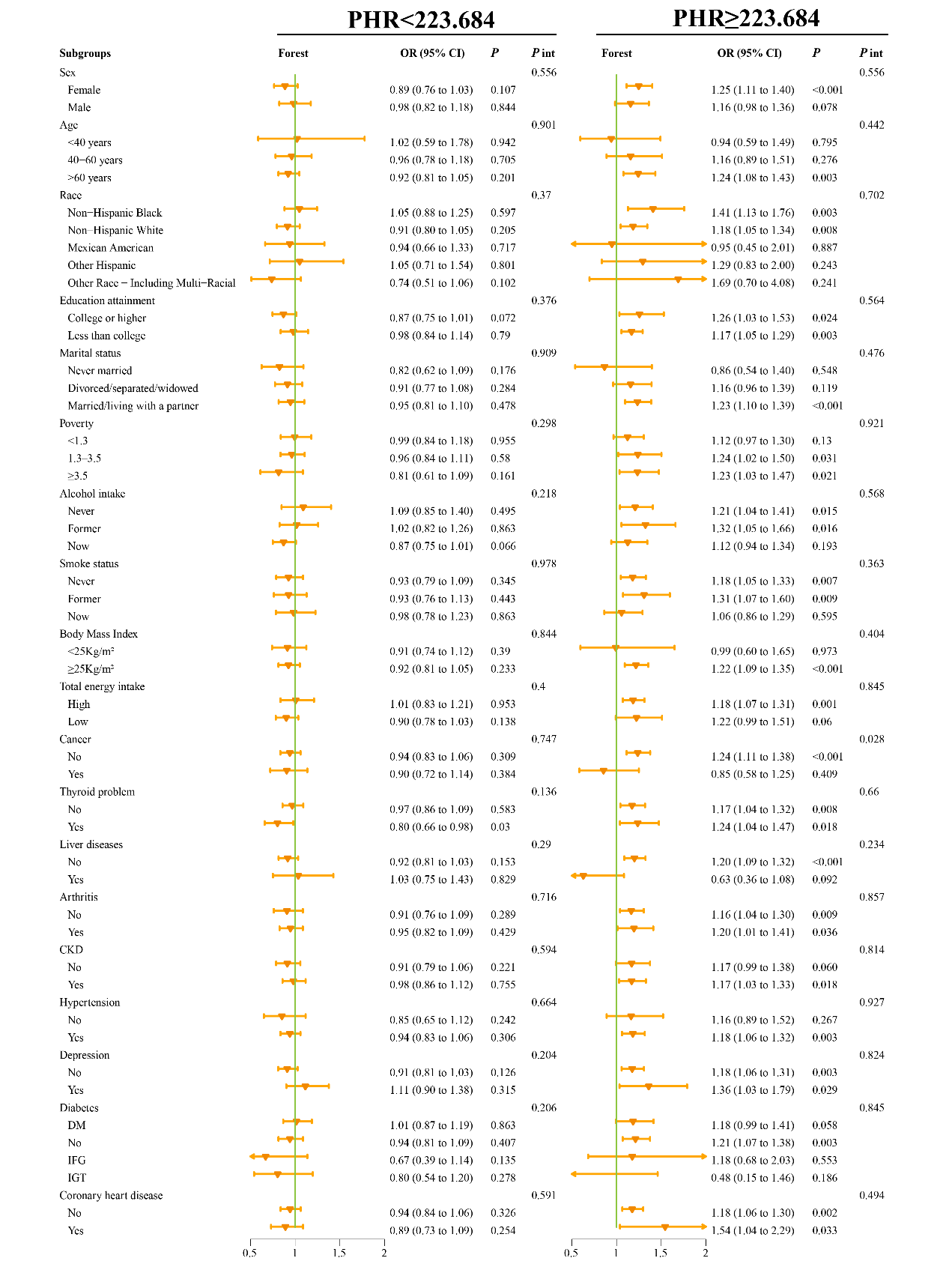


## Supplementary Figure 2: Forest plot of subgroup analysis and interaction tests for the association between PHR and CVD mortality among stroke survivals

**Figure legend:** All models adjusted for 8 risk factors other than stratification variables, and the significance of the interaction was determined by the likelihood ratio test.

**Abbreviation:** PHR, platelet-to-high-density lipoprotein cholesterol ratio; CVD, cardiovascular disease; CKD, chronic kidney disease; HR, hazard ratio; CI, confidence interval; *P* int, *P* for interaction.


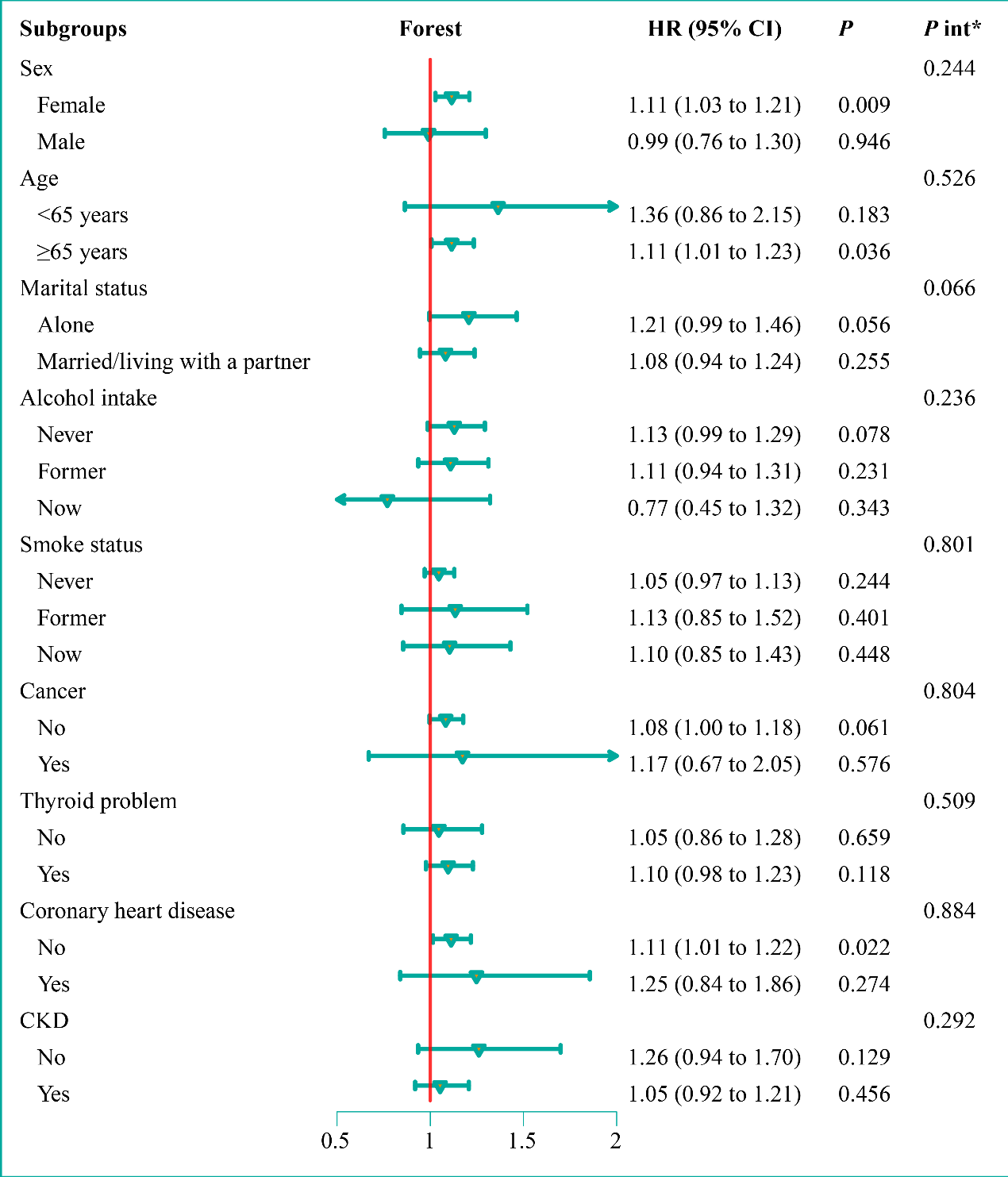


# Supplementary Tables

## Supplementary Table 1: Weighted characteristics of the eligible 812 participants in the analysis of PHR and HDL-C with CVD mortality among stroke survivals

| **Characteristics** | **Total**  **(N=812)** | **Alive**  **(N=673)** | **CVD-specific**  **death (N=139)** | ***P* value** |
| --- | --- | --- | --- | --- |
| **PHR, median(Q1,Q3)** | **182.17(138.13,245.63)** | **183.33(138.21,245.63)** | **177.50(137.62,240.82)** | **0.91** |
| **Platelet (1000 cells/μL), median(Q1,Q3)** | **235.00(192.00,280.00)** | **235.00(194.00,277.00)** | **227.00(176.00,285.00)** | **0.46** |
| **HDL-C (mmol/L), median(Q1,Q3)** | **1.27(1.01,1.55)** | **1.27(1.03,1.60)** | **1.22(1.01,1.50)** | **0.13** |
| **Follow-up time (years), Mean (S.E)** | **7.00(0.19)** | **7.32(0.21)** | **4.83(0.33)** | **< 0.0001** |
| **Age (year), Mean (S.E)** | **62.17(0.68)** | **60.45(0.71)** | **73.85(0.87)** | **< 0.0001** |
| **Sex, n (%)** |  |  |  | **0.03** |
| **Female** | **423(55.28)** | **372(56.98)** | **51(43.76)** |  |
| **Male** | **389(44.72)** | **301(43.02)** | **88(56.24)** |  |
| **Race, n (%)** |  |  |  | **0.09** |
| **Mexican American** | **79(4.70)** | **71(4.92)** | **8(3.16)** |  |
| **Non-Hispanic Black** | **229(14.81)** | **197(15.22)** | **32(12.00)** |  |
| **Non-Hispanic White** | **399(70.00)** | **309(68.41)** | **90(80.78)** |  |
| **Other Hispanic** | **56(3.33)** | **49(3.57)** | **7(1.74)** |  |
| **Other Race - Including Multi-Racial** | **49(7.16)** | **47(7.87)** | **2(2.32)** |  |
| **Education attainment, n (%)** |  |  |  | **0.26** |
| **Less than college** | **462(51.86)** | **380(50.93)** | **82(58.15)** |  |
| **College or higher** | **350(48.14)** | **293(49.07)** | **57(41.85)** |  |
| **Marital status, n (%)** |  |  |  | **0.01** |
| **Never married** | **72(7.48)** | **66(8.04)** | **6(3.72)** |  |
| **Divorced/separated/widowed** | **293(29.07)** | **233(27.40)** | **60(40.43)** |  |
| **Married/living with a partner** | **447(63.44)** | **374(64.56)** | **73(55.86)** |  |
| **Poverty, n (%)** |  |  |  | **0.01** |
| **<1.3** | **319(30.56)** | **275(31.24)** | **44(25.94)** |  |
| **1.3–3.5** | **342(43.00)** | **270(40.95)** | **72(56.90)** |  |
| **>3.5** | **151(26.44)** | **128(27.81)** | **23(17.16)** |  |
| **Alcohol status, n (%)** |  |  |  | **< 0.0001** |
| **Never** | **118(12.68)** | **95(11.58)** | **23(20.13)** |  |
| **Former** | **258(28.48)** | **188(26.04)** | **70(45.03)** |  |
| **Now** | **436(58.84)** | **390(62.38)** | **46(34.84)** |  |
| **Smoke, n (%)** |  |  |  | **0.05** |
| **Never** | **333(43.32)** | **280(43.90)** | **53(39.39)** |  |
| **Former** | **282(33.09)** | **216(31.42)** | **66(44.37)** |  |
| **Now** | **197(23.59)** | **177(24.68)** | **20(16.24)** |  |
| **Body Mass Index (BMI), Mean (S.E)** | **30.76(0.32)** | **30.99(0.36)** | **29.24(0.74)** | **0.04** |
| **Total energy intake (Kcal) Mean (S.E)** | **1912.72(47.85)** | **1943.61(53.32)** | **1703.33(80.55)** | **0.01** |
| **Cancer, n (%)** | **158(19.37)** | **116(17.44)** | **42(32.41)** | **0.02** |
| **Thyroid problem, n (%)** | **165(22.35)** | **141(22.68)** | **24(20.10)** | **0.62** |
| **Liver diseases, n (%)** | **57(6.66)** | **50(7.13)** | **7(3.53)** | **0.17** |
| **Arthritis, n (%)** | **458(55.10)** | **376(54.17)** | **82(61.41)** | **0.2** |
| **CKD, n (%)** | **328(35.95)** | **229(31.44)** | **99(66.51)** | **< 0.0001** |
| **Hypertension, n (%)** | **652(77.00)** | **529(75.56)** | **123(86.77)** | **0.02** |
| **Diabetes, n (%)** |  |  |  | **0.17** |
| **DM** | **321(35.61)** | **259(35.18)** | **62(38.55)** |  |
| **IFG** | **38(6.06)** | **34(6.54)** | **4(2.81)** |  |
| **IGT** | **29(2.90)** | **22(2.48)** | **7(5.75)** |  |
| **No** | **424(55.43)** | **358(55.80)** | **66(52.89)** |  |
| **Depression, n (%)** | **156(18.67)** | **138(18.98)** | **18(16.60)** | **0.54** |
| **Coronary heart disease, n (%)** | **132(17.33)** | **97(15.44)** | **35(30.16)** | **0.002** |
| **Antiplatelet drugs, n (%)** | **191(21.84)** | **141(20.04)** | **50(34.02)** | **0.003** |
| **Statin drugs, n (%)** | **414(51.80)** | **340(51.96)** | **74(50.73)** | **0.81** |

**Abbreviation:** HDL-C, high-density lipoprotein cholesterol; PHR, platelet-to-high-density lipoprotein cholesterol ratio; CVD, cardiovascular disease; CKD, chronic kidney disease; IFG, impaired fasting glycaemia; IGT, impaired glucose tolerance; DM, diabetes; SE, standard error.

## Supplementary Table 2: Sensitivity analysis for the association between PHR and the odds of stroke

| **Further adjusted for antiplatelet drug and statin drugs in Model 3** | | | | | | | | | |
| --- | --- | --- | --- | --- | --- | --- | --- | --- | --- |
| **PHR** |  | Model 0 | | Model 1 | | Model 2 | | Model 3^a^ | |
| Overall |  | OR (95% CI) | *P* | OR (95% CI) | *P* | OR (95% CI) | *P* | OR (95% CI) | *P* |
|  | Per SD increase | 1.03(0.93,1.15) | 0.55 | 1.23(1.13,1.33) | <0.0001 | 1.17(1.07,1.27) | <0.001 | 1.11(1.01,1.22) | 0.04 |
|  | Per 100 increase | 1.04(0.91,1.19) | 0.55 | 1.29(1.16,1.42) | <0.0001 | 1.21(1.09,1.34) | <0.001 | 1.13(1.01,1.27) | 0.04 |
|  |  |  |  |  |  |  |  |  |  |
| PHR<223.684 | Per SD increase | 0.87(0.78,0.97) | 0.01 | 1.02(0.92,1.14) | 0.65 | 0.98(0.89,1.09) | 0.77 | 0.94(0.84,1.05) | 0.25 |
|  | Per 100 increase | 0.70(0.54,0.91) | 0.01 | 1.06(0.81,1.39) | 0.65 | 0.96(0.74,1.26) | 0.77 | 0.85(0.64,1.12) | 0.25 |
|  |  |  |  |  |  |  |  |  |  |
| PHR≥223.684 | Per SD increase | 1.22(1.12,1.33) | <0.0001 | 1.26(1.14,1.38) | <0.0001 | 1.22(1.12,1.34) | <0.0001 | 1.18(1.06,1.31) | 0.002 |
|  | Per 100 increase | 1.31(1.17,1.47) | <0.0001 | 1.36(1.19,1.55) | <0.0001 | 1.32(1.16,1.49) | <0.0001 | 1.25(1.09,1.44) | 0.002 |
| **Without weighted** | | | | | | | | | |
| **PHR** |  | Model 0 | | Model 1 | | Model 2 | | Model 3^b^ | |
| Overall |  | OR (95% CI) | *P* | OR (95% CI) | *P* | OR (95% CI) | *P* | OR (95% CI) | *P* |
|  | Per SD increase | 1.01(0.95,1.08) | 0.68 | 1.21(1.14,1.28) | <0.0001 | 1.16(1.09,1.22) | <0.0001 | 1.10(1.04,1.18) | 0.002 |
|  | Per 100 increase | 1.02(0.94,1.09) | 0.68 | 1.26(1.17,1.35) | <0.0001 | 1.19(1.11,1.28) | <0.0001 | 1.13(1.05,1.22) | 0.002 |
|  |  |  |  |  |  |  |  |  |  |
| PHR<223.684 | Per SD increase | 0.90(0.83,0.97) | 0.004 | 1.04(0.97,1.13) | 0.26 | 1.01(0.94,1.09) | 0.82 | 0.96(0.89,1.04) | 0.34 |
|  | Per 100 increase | 0.76(0.63,0.91) | 0.004 | 1.12(0.92,1.35) | 0.26 | 1.02(0.84,1.24) | 0.82 | 0.91(0.74,1.11) | 0.34 |
|  |  |  |  |  |  |  |  |  |  |
| PHR≥223.684 | Per SD increase | 1.18(1.09,1.27) | <0.0001 | 1.22(1.12,1.33) | <0.0001 | 1.21(1.11,1.31) | <0.0001 | 1.18(1.07,1.28) | <0.001 |
|  | Per 100 increase | 1.25(1.12,1.39) | <0.0001 | 1.32(1.17,1.48) | <0.0001 | 1.29(1.15,1.45) | <0.0001 | 1.25(1.10,1.41) | <0.001 |

**Notes:**

Model 0: Not adjusted;

Model 1: Adjusted for age, sex, education attainment, and ethnicity;

Model 2: Further adjusted for marital status, poverty-income ratio, smoking, and drinking status, BMI, and total energy intake based on Model 1;

Model 3^a^: Further adjusted for arthritis, thyroid problems, cancer, diabetes, depression, hypertension, liver diseases, CHD, CKD, **antiplatelet drug and statin drugs** based on Model 2.

Model 3^b^: Further adjusted for arthritis, thyroid problems, cancer, diabetes, depression, hypertension, liver diseases, CHD, and CKD based on Model 2.

**Abbreviations**: HDL-C, high-density lipoprotein cholesterol; PHR, platelet-to-high-density lipoprotein cholesterol ratio; BMI, body mass index; CHD, coronary heart disease; CKD, chronic kidney disease; SD, standard deviation; OR, odds ratio; CI, confidence interval.

## Supplementary Table 3: Sensitivity analysis for the association between PHR and the odds of CVD mortality among stroke survivors

| **Further adjusted for antiplatelet drug and statin drugs in Model 3** | | | | | | | | | |
| --- | --- | --- | --- | --- | --- | --- | --- | --- | --- |
| **PHR** | Model 0 | | Model 1 | | Model 2 | | Model 3^a^ | | |
|  | HR (95% CI) | *P* | HR (95% CI) | *P* | HR (95% CI) | *P* | HR (95% CI) | *P* |  |
| Per SD increase | 1.07(0.93,1.23) | 0.33 | 1.11(1.03,1.19) | 0.01 | 1.10(1.02,1.18) | 0.01 | 1.10(1.02,1.19) | 0.01 |  |
| Per 100 increase | 1.07(0.94,1.21) | 0.33 | 1.10(1.03,1.18) | 0.01 | 1.10(1.02,1.17) | 0.01 | 1.09(1.02,1.17) | 0.01 |  |
| **Without weighted** | | | | | | | | |  |
| **PHR** | Model 0 | | Model 1 | | Model 2 | | Model 3^b^ | |  |
|  | HR (95% CI) | *P* | HR (95% CI) | *P* | HR (95% CI) | *P* | HR (95% CI) | *P* |  |
| Per SD increase | 1.00(0.86,1.16) | 0.99 | 1.09(0.99,1.22) | 0.09 | 1.10(0.98,1.23) | 0.10 | 1.13(1.00,1.27) | 0.04 |  |
| Per 100 increase | 1.00(0.87,1.15) | 0.99 | 1.09(0.99,1.20) | 0.09 | 1.09(0.98,1.22) | 0.10 | 1.12(1.00,1.25) | 0.04 |  |

**Notes:**

Model 0: Not adjusted;

Model 1: Adjusted for age, sex;

Model 2: Further adjusted for marital status, smoking, and drinking status based on Model 1;

Model 3^a^: Further adjusted for thyroid problems, cancer, CHD, CKD, **antiplatelet drug, and statin drugs** based on Model 2.

Model 3^b^: Further adjusted for arthritis, thyroid problems, cancer, diabetes, depression, hypertension, liver diseases, CHD, and CKD based on Model 2.

**Abbreviations**: HDL-C, high-density lipoprotein cholesterol; PHR, platelet-to-high-density lipoprotein cholesterol ratio; CVD, cardiovascular disease; CHD, coronary heart disease; CKD, chronic kidney disease; SD, standard deviation; HR, hazard ratio; CI, confidence interval.
